# Genome‐Wide DNA Methylation and Copy Number Alterations in Gastrointestinal Stromal Tumors

**DOI:** 10.1002/gcc.70046

**Published:** 2025-03-27

**Authors:** Tony G. Kleijn, Baptiste Ameline, Roos F. Bleckman, Wierd Kooistra, Evert van den Broek, Gilles F. H. Diercks, Bettien M. van Hemel, Bert Timmer, Wim Timens, Gursah Kats‐Ugurlu, Léon C. van Kempen, Boudewijn van Etten, Ed Schuuring, Albert J. H. Suurmeijer, Jacco J. de Haan, Daniel Baumhoer, Anna K. L. Reyners, Arjen H. G. Cleven

**Affiliations:** ^1^ Department of Pathology and Medical Biology University Medical Center Groningen, University of Groningen Groningen the Netherlands; ^2^ Bone Tumor Reference Center at the Institute for Medical Genetics and Pathology, University Hospital Basel, University of Basel Basel Switzerland; ^3^ Department of Medical Oncology University Medical Center Groningen, University of Groningen Groningen the Netherlands; ^4^ Department of Pathology Antwerp University Hospital, University of Antwerp Edegem Belgium; ^5^ Department of Surgery University Medical Center Groningen, University of Groningen Groningen the Netherlands; ^6^ Department of Pathology Amsterdam University Medical Center Amsterdam the Netherlands

**Keywords:** copy number variation, DNA methylation, gastrointestinal stromal tumor (GIST), profiling

## Abstract

Gastrointestinal stromal tumors (GISTs) span a broad clinical spectrum, from indolent neoplasms to life‐threatening metastatic tumors. A persistent limitation of current risk stratification systems is that a subset of GISTs is graded as low‐risk but nevertheless metastasizes. Therefore, new predictive factors that improve risk stratification are needed. In this exploratory study, we investigated the potential of genome‐wide DNA methylation profiling and copy number variation (CNV) analysis as additional prognostic tools for GISTs. We collected a cohort of 28 patients with GIST diagnosed between 2001 and 2022, with available follow‐up and molecular data. This included 15 patients without progressive disease (seven low‐risk and eight moderate‐ to high‐risk GISTs) and 13 with progressive disease. Among those with progression, eight experienced recurrence or metastasis post‐surgery (one low‐risk, seven high‐risk GISTs), while five had metastatic disease at initial diagnosis. Risk stratification was determined according to Miettinen's criteria. Genome‐wide DNA methylation data and CNV plots were generated from imatinib‐naïve primary GISTs using the Illumina Infinium MethylationEPIC BeadChip array. Unsupervised cluster analysis revealed distinct DNA methylation patterns predominantly associated with anatomical location and genotype. Differential DNA methylation analysis comparing primary gastric GISTs associated with and without progressive disease showed 8 differentially methylated regions spanning the coding and promoter areas of 6 genes. CNV analysis demonstrated that GISTs associated with progressive disease had the most CNVs, whereas low‐risk, non‐progressive GISTs had the fewest. Despite the limited sample size, this exploratory study indicates that genome‐wide DNA methylation profiling and CNV analysis could enhance GIST risk stratification.

## 1 Introduction

Gastrointestinal stromal tumor (GIST) is the most common mesenchymal neoplasm of the gastrointestinal tract, with approximately 65% of cases occurring in the stomach [1]. GISTs span a broad clinical spectrum ranging from indolent neoplasms to life‐threatening tumors with metastatic spread. Various risk stratification systems have been published to predict the malignant potential of GISTs [2, 3, 4]. The primary criteria currently used for risk assessment include tumor location, size, mitotic index, and tumor rupture [5]. For patients with surgically resected GIST who are considered high‐risk for recurrence, the standard of care includes at least 3 years of adjuvant imatinib therapy, which has been shown to enhance both recurrence‐free survival and overall survival [6, 7, 8, 9].

Most GISTs are caused by gain‐of‐function mutations in type III receptor tyrosine kinases *KIT* (accounting for 75%–80%) or *PDGFRA* (< 10%), both located on chromosome 4q [10]. These mutations lead to constitutive activation of signaling pathways that promote proliferation, inhibit apoptosis, and regulate cell differentiation, adhesion, and motility [11]. Besides *KIT* and *PDGFRA* mutations, GISTs can also involve other genetic alterations, including mutations in *NF1*, *BRAF*, *KRAS*, and *PIK3CA*, as well as deficiencies in succinate dehydrogenase (SDH) [12].

The prognosis for patients with GIST is influenced by the specific mutation type, with *KIT* mutations generally associated with a worse prognosis than *PDGFRA* mutations. Importantly, the response to imatinib treatment correlates with the specific activating mutation in *KIT* or *PDGFRA*. Tumors harboring *KIT* exon 11 mutations are generally more responsive to imatinib than those lacking *KIT* and *PDGFRA* mutations [13]. While most *PDGFRA*‐mutated GISTs are sensitive to imatinib, the p.D842V variant is resistant [14]. Furthermore, patients with advanced GIST harboring *KIT* exon 9 mutations have better disease‐free survival when treated with a doubledose of imatinib, which is therefore considered the standard treatment for this subgroup [15].

The progression from microscopic preclinical GIST to clinically manifest tumors with high malignant potential involves stepwise inactivation of tumor suppressor genes. For example, specific chromosomal regions are frequently lost in GISTs (except for the SDH‐deficient ones), including 1q, 14q, 15q, and 22q, which involve genes, such as MYC‐associated factor X (*MAX*), protein phosphatase 1A (*PPM1A*), and neurofibromin 2 (*NF2*) [10, 16, 17]. In addition to genetic changes, epigenetic factors, such as DNA methylation, histone modifications, small non‐coding RNAs, and chromatin remodeling, also contribute to GIST pathogenesis [18]. Genome‐wide DNA methylation analyses have revealed that methylation accumulates throughout the genome during GIST progression and may contribute to disease progression [19]. Notably, hypermethylation of *CDKN2A, REC8*, *PAX3*, and *SPP1* has been associated with aggressive behavior and unfavorable prognoses [19, 20, 21].

In clinical practice, discrepancies often arise between the predicted risk of GISTs and their actual clinical course. For example, some GISTs graded as low‐risk still metastasizes [3]. Therefore, new predictive factors that improve risk stratification in GISTs are needed. In recent years, DNA methylation profiling and copy number variation (CNV) analysis have emerged as diagnostic and prognostic tools in various cancers [22, 23]. In this exploratory study, we investigated the potential of genome‐wide DNA methylation profiling and CNV analysis as additional prognostic tools for GIST.

## 2 Materials and Methods

### 2.1 Tumor Samples

Sufficient well‐preserved primary imatinib‐naïve tumor tissue from 28 GIST cases, with available molecular and follow‐up data, was retrieved from the pathology department archive at the University Medical Center Groningen (UMCG), an expert sarcoma center in the Netherlands. The cohort included 15 patients without progressive disease (seven low‐risk and eight moderate‐ to high‐risk GISTs) and 13 with progressive disease. Among those with progression, eight experienced recurrence or metastasis post‐surgery (one low‐risk and seven high‐risk GISTs), while five had metastatic disease at initial diagnosis. For this exploratory study, we retrieved 28 patients to ensure a minimum of seven cases per group (low‐risk vs. moderate‐ to high‐risk vs. progressive disease) to reduce potential clustering artifacts [23]. The patients were diagnosed at our institution between 2001 and 2022. Risk grades were determined on primary imatinib‐naïve tumor tissue using the Armed Forces Institute of Pathology (AFIP) criteria (Miettinen's criteria) during diagnostic work‐up [24]. Disease progression was defined according to the revised RECIST (Response Evaluation Criteria in Solid Tumors) guidelines (version 1.1). Two expert soft tissue tumor pathologists (A.C. and A.S.) reviewed histology, immunohistochemistry, and molecular findings to confirm each diagnosis. Clinical data, including patient age, sex, primary tumor site and size, tumor rupture, risk grade, treatment, and clinical outcome, were obtained from the electronic patient record system.

Tumor samples were obtained from the archive with ethical board approval (UMCG RR202200287) and coded (pseudonymized) according to the Code of Conduct for Health Research (www.coreon.org).

### 2.2 DNA Extraction and Genome‐Wide Methylation Data Generation

Genomic DNA was extracted from fresh frozen (*n* = 27) or formalin‐fixed paraffin‐embedded (FFPE, *n* = 1) biopsy or resection specimens, using only representative tumor tissue to minimize methylation contamination from normal tissue. Tumor purity ranged from 60% to 97% (median = 95%). For fresh frozen tissue, DNA extraction involved a salt/chloroform‐based protocol, while DNA was extracted from FFPE tissue using the QIAamp DNA FFPE Tissue Kit (Qiagen, Hilden, Germany) according to the manufacturer's instructions. DNA was quantified using a Qubit Fluorometer. More than 250 ng of genomic DNA was obtained from each tumor for array‐based methylation analysis. Genome‐wide DNA methylation data were generated using the Illumina Infinium Human MethylationEPIC v1.0 BeadChip, which covered approximately 850 000 cytosine‐guanine (CpG) sites across the genome.

### 2.3 Heidelberg Sarcoma Methylation Classifier

Raw intensity data files (IDATs) were uploaded to the German Cancer Research Center's (DKFZ) DNA methylation‐based sarcoma classifier (version 12; www.molecularsarcomapathology.org) for diagnostic validation. The classifier provided a suggested methylation class along with a corresponding calibrated score for each sample. The calibrated score represents the probability of confidence for the assigned methylation class. As defined by Koelsche et al. the classifier was considered to have made a successful prediction if the sample received a calibrated score of 0.9 or higher [23].

### 2.4 Methylation Array Process

Raw IDATs from our cohort were combined with available external cases (*n* = 40) [23, 25], resulting in a total data set of 68 GISTs. For the 40 external GISTs, demographic information (age and sex), tumor location, and in three cases mutation status were available (risk grades and follow‐up data were not available). Clinical details for all cases are provided in Table S1.

Raw methylation data from the MethylationEpic BeadChips were processed using the R package “minfi” (https://bioconductor.org/packages/minfi/). Probes associated with single‐nucleotide polymorphisms (SNPs), non‐CpG islands, and sex chromosomes were excluded from the analysis. Samples with a mean detection *p* value of > 0.01 were removed. Prior to generating dimension reduction visualizations, the “preprocessQuantile” function was used, while the “preprocessIllumina” function was used before deriving CNV plots. Batch effect correction was applied to the beta values using the R package “ChAMP” (https://bioconductor.org/packages/ChAMP/) to eliminate potential bias related to sample type (FFPE vs. fresh frozen).

### 2.5 Unsupervised Cluster Analysis

To explore DNA methylation patterns, unsupervised cluster analysis was performed using the top 5000 most differentially methylated CpG sites. A non‐linear dimension reduction method, Uniform Manifold Approximation and Projection (UMAP), was applied to the results of a principal component analysis (PCA) calculated via singular value decomposition of the beta methylation matrix. Graph generation was performed using the R package “uwot” (https://github.com/jlmelville/uwot). The UMAP model was generated using the following settings: PCA = 10, neighbors = 6, with default settings for all remaining parameters.

### 2.6 Differential Methylation Analysis

Differential DNA methylation analysis was performed to identify significantly differentially methylated CpG sites and regions by comparing all 850 000 CpG sites between progressive (*n* = 3) versus those without progressive disease (*n* = 13). Only gastric GISTs were selected to minimize confounding effects due to tumor localization. Raw data were preprocessed as described above. The R package “DMRcate” (https://github.com/rcavalcante/DMRcate/) was loaded, and the function “cpg.annotate” was applied on the *M* values matrix using the default parameters. Differentially methylated regions (DMRs) were identified using the function “dmrcate” with a minimum of 3 differentially methylated CpG islands and a maximal distance between two CpG islands of 500 base pairs. Each differently methylated region was labeled using the R package “annotatr” in order to identify proximal promoters of protein‐coding genes.

### 2.7 CNV Analysis

Copy number plots were derived from the methylation data using the R package “conumee” (http://bioconductor.org/packages/conumee/). Data preprocessing followed the same steps as described above. Copy number segmentation settings were as follows: a minimum of 25 probes per bin and a minimum bin size of 50 000 base pairs. CNVs were considered significant if a minimum of 5 bins were present. Each CNV plot was manually examined. To identify statistically significant recurrent CNVs, GISTIC2, and the R package “CNsummaryplots” were employed (https://broadinstitute.github.io/gistic2/, https://github.com/dstichel/CNsummaryplots). The segmentations generated by “conumee” served as input for GISTIC2 and “CNsummaryplots.” In GISTIC2, CNV events were considered significant if they had a False Discovery Rate *q* value < 0.05 at a 90% confidence level. Fisher's exact test was used to assess significant differences in CNV frequencies between groups.

## 3 Results

### 3.1 Clinicopathologic Characteristics

Our cohort consisted of 28 patients with a median age at presentation of 64 years (range: 19–88 years), including 14 males (Table 1). Tumors had mutations in *KIT* (*n* = 23), *PDGFRA* (*n* = 4), and *SDHA* (*n* = 1), with no wild‐type cases. Primary tumors were located in the stomach (*n* = 17), small intestine (*n* = 10), and rectum (*n* = 1). Among the gastric GISTs, tumor sites included the fundus (*n* = 5), corpus (*n* = 8), and antrum (*n* = 4). Tumor sizes ranged from 3 to 23 cm (median 7.5 cm). Based on Miettinen's criteria, primary tumors were graded as low‐risk (*n* = 8), moderate‐risk (*n* = 2), and high‐risk (*n* = 13). Fifteen patients (seven low‐risk and eight moderate‐ to high‐risk GISTs) remained disease‐free after surgery with a 7.2‐year median follow‐up (range: 45–238 months). Conversely, 13 patients had progressive disease. One patient with low‐risk GIST (untreated with adjuvant imatinib) and seven patients with high‐risk GIST (treated with adjuvant imatinib) experienced recurrence or metastasis post‐surgery after a median follow‐up of 1.7 years (range: 1–52 months). Five patients (cases no. 17, 18, 19, 23, and 28) had metastatic disease at initial diagnosis; their tumors were not formally graded but would have been graded as moderate‐risk (*n* = 3) and high‐risk (*n* = 2).

**TABLE 1:** Clinical pathological features and mutational status.

No	Tumor site	Tumor size (cm)	Mitotic count/(5 mm^2^)	Risk grade (progression %)	Driver gene	Amino acid change by driver gene mutation	Tumor rupture	Imatinib	Disease‐free survival (months)	Survival (months)
1	Stomach (fundus)	3	< 5	Very low (1.9%)	*KIT*	E11: p.E554_V559del	No	No	NED (56)	DOC (56)
2	Stomach (fundus)	4	1	Very low (1.9%)	*PDGFRA*	p.D842V	No	No	NED (105)	Alive (105)
3	Stomach (corpus)	5	3	Very low (1.9%)	*KIT*	E11: p.W557R	No	No	NED (121)	Alive (121)
4	Stomach (corpus)	4	2	Very low (1.9%)	*KIT*	E11: p.K550_M552del	No	No	NED (66)	Alive (66)
5	Stomach (fundus)	4	4	Very low (1.9%)	*KIT*	E11: p.Q556_V560delinsH	No	No	NED (64)	Alive (64)
6	Stomach (fundus)	4	3	Very low (1.9%)	*KIT*	E11: p.K558_D752delinsN	No	No	NED (118)	Alive (118)
7	Stomach (antrum)	3	< 5	Very low (1.9%)	*KIT*	E11: p.V560D	No	No	NED (45)	DOC (45)
8	Stomach (antrum)	3	17	Moderate (16%)	*KIT*	E11: p.P577_L589dup	No	No	NED (66)	Alive (66)
9	Stomach (corpus)	12	3	Moderate (10%)	*PDGFRA*	p.D842V	No	No	NED (94)	Alive (94)
10	Stomach (corpus)	7	7	High (55%)	*PDGFRA*	p.D842V	No	ADJ	NED (130)	Alive (130)
11	Stomach (antrum)	8	6	High (55%)	*PDGFRA*	p.D842V	No	No	NED (94)	Alive (94)
12	Stomach (corpus)	7	12	High (55%)	*KIT*	E11: p.V560D	Yes	ADJ	NED (73)	Alive (73)
13	Stomach (fundus)	5	12	High (55%)	*KIT*	E11: p.D579del	No	ADJ	NED (86)	Alive (86)
14	Small intestine	13	< 5	High (34%)	*KIT*	E11	Yes	ADJ	NED (238)	Alive (238)
15	Ileum	7	> 5	High (86%)	*KIT*	E11: p.V555_Q556del	No	ADJ	NED (75)	Alive (75)
16	Stomach (corpus)	9	2	Low (3.6%)	*KIT*	E9: p.A502_Y503dup	No	No	PD (21): LN	DOC (47)
17	Small intestine	5	> 5	Not graded	*KIT*	E11: p.N573_V583ins	Yes	ADJ	PD (0): ABD	DOD (73)
18	Small intestine	7	2	Not graded	*KIT*	E11: p.L576P	Yes	ADJ	PD (0): PS	Alive (31)
19	Stomach (corpus)	10	> 5	Not graded	*KIT*	E11: p.K558_P573delinsT	No	ADJ	PD (0): ABD	Alive (111)
20	Stomach (corpus)	20	> 5	High (86%)	*KIT*	E11	No	ADJ	PD (42): Re + HEP	DOD (101)
21	Ileum	10	30	High (86%)	*KIT*	E9: p.A502_Y503dup	Yes	NADJ + ADJ	PD (5): PS	DOD (36)
22	Small intestine	20	24	High (86%)	*KIT*	E11: p.K555_W557delinsNHCT	No	ADJ	PD (37): HEP + PER	Alive (44)
23	Jejunum	21	1	Not graded	*KIT*	E11	Yes	ADJ	PD (0): HEP	DOD (46)
24	Ileum	18	11	High (86%)	*KIT*	E11: p.W557R	Yes	ADJ	PD (20): HEP	Alive (149)
25	Ileum	23	> 5	High (86%)	*KIT*	E11	No	ADJ	PD (1): HEP	DOD (62)
26	Ileum	12	> 5	High (86%)	*KIT*	E11: p.V559D	Yes	ADJ	PD (52): Re	Alive (139)
27	Rectum	12	> 5	High (Unknown)	*KIT*	E11	No	ADJ	PD (9): HEP	DOD (59)
28	Stomach	20	3	Not graded	*SDHA*	p.Arg75Ter	No	No	PD (0): LN	Alive (17)

Abbreviations: ABD, intra‐abdominal metastasis; ADJ, adjuvant; DOC, died of other cause; DOD, died of disease; HEP, hepatic metastasis; LN, lymph node metastasis; NADJ, neoadjuvant; NED, no evidence of disease after surgery; PD, progressive disease; PER, peritoneal metastasis; PS, peritoneal sarcomatosis; Re, recurrence.

### 3.2 DNA Methylation Analysis

All 28 primary imatinib‐naïve GIST samples yielded interpretable DNA methylation data. The Heidelberg sarcoma classifier successfully identified 24 cases as GIST with calibrated scores > 0.9, indicating high‐confidence classification (Table S1). Four cases had scores below 0.9: Three *KIT*‐mutated GISTs with a lower tumor percentage had calibrated scores between 0.3 and 0.9, while one *SDH*‐deficient GIST received no match (calibrated score < 0.3).

**FIGURE 1:**
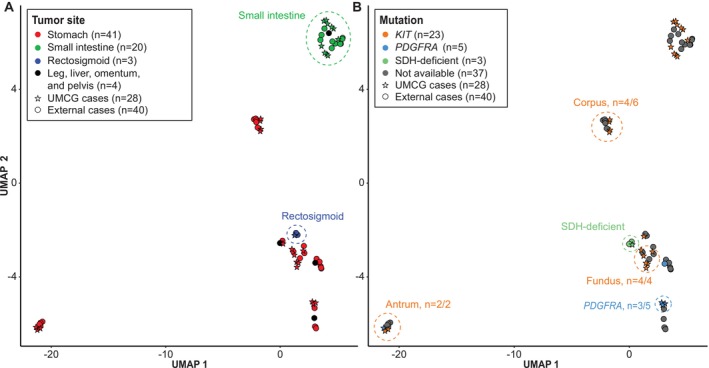
Methylation‐based clustering. Unsupervised cluster analysis of 68 gastrointestinal stromal tumors (GISTs) revealed distinct DNA methylation patterns associated with anatomical location (A) and genotype (B). GISTs from the stomach and small intestine formed separate clusters, with rectosigmoid GISTs clustering separate near gastric GISTs. The 12 *KIT*‐mutated gastric GISTs could be further subdivided by their location in the stomach (antrum, corpus, and fundus). Of five *PDGFRA*‐mutated GISTs, three formed a distinct cluster, while the antral *PDGFRA*‐mutated GIST clustered with other antral cases. The three SDH‐deficient GISTs clustered separately from *KIT*‐ and *PDGFRA*‐mutated GISTs.

Unsupervised cluster analysis of 68 GISTs (i.e., 28 GISTs from the current study, and 40 external GISTs for which only age, sex, tumor location, and in three cases mutation status were available) revealed a predominant DNA methylation clustering pattern based on anatomical location (stomach vs. small intestine vs. rectosigmoid; Figure 1A) and genotype (*KIT* vs. *PDGFRA* vs. SDH‐deficient; Figure 1B). Interestingly, our included *KIT*‐mutated gastric GISTs could be subdivided into antrum, corpus, and fundus sub‐localization.

Unsupervised cluster analysis using solely our 28‐patient cohort (with full clinical and molecular data) also demonstrated a predominant DNA methylation clustering pattern based on anatomical localization and genotype that did not appear to be influenced by risk grades (low‐risk vs. high‐risk; data not shown).

Differential DNA methylation analysis comparing *KIT*‐ and *PDGFRA*‐mutated gastric GISTs associated with progressive disease versus non‐progressive cases identified 134 significant differentially methylated CpG sites (*p* value < 0.05; mean diff > 0.1) and 8 differentially methylated regions spanning the coding and promoter areas of 6 genes. Compared to non‐metastatic tumors, the metastatic GISTs exhibited hypomethylation in the promoter region/transcription start site of *CLCN3* (Figure S1), *TNXB*, *PRRT1*, and *ELOVL2‐AS1*, and hypermethylation in *COX4I2* and *SYCP2L*.

Given their reported association with GIST aggressiveness and unfavorable prognosis, the methylation levels of *CDKN2A*, *PAX3*, *REC8*, and *SPP1* were analyzed [19, 20, 21]. While some GIST cases associated with progressive disease showed increased methylation levels of *PAX3* and *REC8* (Figure S2), no significant differences were observed in the average methylation levels of these four genes between the low‐risk, high‐risk, and progressive GIST groups.

### 3.3 CNV Analysis

**FIGURE 2:**
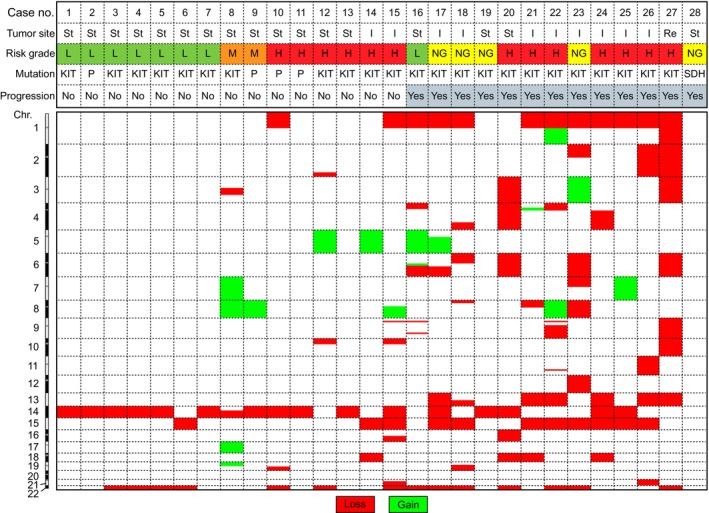
Summary of copy number variations (CNVs) in UMCG cohort of gastrointestinal stromal tumors. H, high‐risk; I, small intestines; L, low‐risk; M, moderate‐risk; NG, not graded; P, *PDGFRA*‐mutated; Re, rectum; St, stomach.

All *KIT*‐ and *PDGFRA*‐mutated GIST samples (*n* = 27) showed CNVs (Figure 2). The majority of CNVs involved losses of entire chromosomes or chromosome arms. Losses occurred approximately six times more frequently than gains. The most frequent chromosomal losses were 1p (44%), 14q (63%), 15q (41%), and 22q (52%). Less frequent losses included 2p, 6, 13q, and 18. Chromosomal gains were identified in 11 cases (41%), primarily affecting 5q (*n* = 4) and 8q (*n* = 4). The *SDHA*‐mutated GIST was the only case with a flat CNV profile.

Significant differences in the frequencies of chromosomal losses were observed between gastric and small intestine GISTs. Losses of 1p occurred in 12.5% of gastric GISTs versus 90% of small intestine GISTs (*p* = 0.035), 14q in 81% of gastric GISTs versus 40% of small intestine GISTs (*p* = 0.046), and 15 in 6% of gastric GISTs versus 100% of small intestine GISTs (*p* = 0.009; Figure S3 A,B). Consequently, certain tumor suppressor genes were more frequently deleted in small intestine GISTs, including *CDKN2C* and *KIF1B* (1p) and *MAP2K1* (15q), whereas *MAX* and *PPM1A* (14q) were more commonly lost in gastric GISTs. Additionally, CNV‐complex tumors (defined as those with > 5 chromosomal changes) were predominantly non‐gastric (19% of gastric GISTs vs. 80% of small intestine GISTs; *p* = 0.004).

**FIGURE 3:**
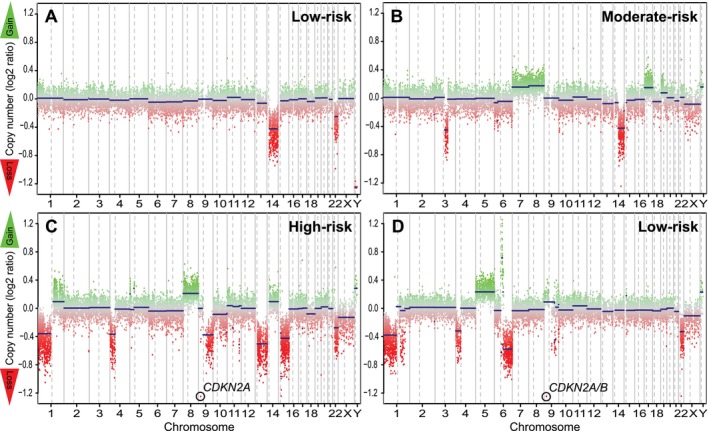
Representative copy number plots. (A) Low‐risk gastrointestinal stromal tumor (GIST; case #3) with loss of chromosomes 14 and 22. (B) Moderate‐risk GIST (case #8) with partial losses of chromosomes 3 and 14, and gains of chromosomes 7, 8, 17, and 19. (C) High‐risk GIST (case #22) showed heterogeneous losses and gains, including homozygous deletion of *CDKN2A*. The patient developed liver and peritoneal metastases 49 months post‐surgery. (D) Low‐risk GIST (case #16) showed multiple losses and gains, including loss of chromosome 1p, focal amplification of chromosome 6, and homozygous deletion of *CDKN2A/B*. The patient developed lymph node metastases 21 months post‐surgery.

The burden of CNVs varied between progressive and non‐progressive GISTs. Low‐risk, non‐progressive GISTs (*n* = 7) had only one or two CNVs (mean 1.6; Figure 3A), with 14q loss as the sole detected abnormality in three cases. Moderate‐ to high‐risk, non‐progressive GISTs (*n* = 8) exhibited a higher number of CNVs (mean 3.8, range 1–8; Figure 3B), while the highest CNV burden (mean 5.9, ranging from 1 to 10) was found in GISTs associated with progressive disease (*n* = 12; Figure 3C). The frequencies of CNVs across different groups, categorized by genotype, risk‐grade, and location, are summarized in Figure S3.

Chromosomal losses in 1p, 2p, 4q, 6q, 9p, 13, 15q, and 18 were frequently observed in GISTs associated with progressive disease, but rarely found in localized, non‐progressive GISTs. In particular, losses of 2p, 4q, and 6q were exclusively identified in the progressive GISTs. As a result, several tumor suppressor genes were frequently lost in progressive GISTs, including *CDKN2C* and *KIF1B* (1p), *DNMT3A* (2p), *CCND3* (6q), *CDKN2A/B* (9p), *RB1* (13q), *MAP2K1* (15q), and *SMAD4* (18q).

Interestingly, the copy number profile of the primary tumor of patient #16, who was diagnosed with a low‐risk GIST and later developed metastatic disease, exhibited, in contrast to the other low‐risk GISTs, multiple chromosomal alterations, including loss of 1p, focal amplification of chromosome 6, and homozygous deletion of *CDKN2A/B* (Figure 3D).

## 4 Discussion

GISTs span a broad clinical spectrum, ranging from indolent tumors that never recur or metastasize to aggressive tumors that become metastatic and result in mortality within a few years. This exploratory study showed that genome‐wide DNA methylation profiling and CNV analysis could be promising additional prognostic tools to improve current risk stratification in patients with GIST.

Our study identified significantly differentially methylated regions within the coding and promoter regions of six genes (*CLCN3*, *COX4I2*, *TNXB*, *PRRT1*, *SYCP2L*, and *ELOVL2‐AS1*) between progressive and non‐progressive gastric GISTs. Although these genes are not well‐established cancer drivers, some have been associated with tumor progression. *CLCN3*, which encodes chloride voltage‐gated channel 3 (CIC‐3), has been implicated in cell proliferation, migration, and chemotherapy resistance in several cancers [26, 27, 28]. Deng et al. found significantly elevated ClC‐3 levels in moderately‐ and poorly differentiated chondrosarcomas, suggesting its involvement in chondrosarcoma pathogenesis [29]. Du et al. reported *CLCN3* upregulation in osteosarcoma cells with high metastatic potency, further supporting its role in tumor progression [30]. In our study, *CLCN3* was hypomethylated in metastatic GISTs, which likely correlates with increased gene expression, aligning with findings in chondrosarcoma and osteosarcoma. Another notable finding was hypomethylation of *TNXB* in metastatic GISTs. Vargas et al. recently reported *TNXB* hypomethylation in metastatic synovial sarcoma compared to primary tumor tissue, in line with our findings [31]. While specific studies on Tenascin X (the protein encoded by *TNXB*) in GISTs are limited, research on a related extracellular matrix glycoprotein, Tenascin C (TNC), has shown that TNC expression is associated with gastric GIST progression and prognosis [32]. This suggests that extracellular matrix components, such as tenascins, may contribute to GIST progression. However, further research is needed to confirm the altered gene expression of these genes through additional gene expression analyses, such as RT‐qPCR or immunohistochemistry (IHC), and to explore their potential role in GIST pathogenesis.

Unsupervised DNA methylation analyses revealed differences in clustering predominantly based on anatomical localization and genotype. These observations are consistent with previous studies [20, 33]. Interestingly, *KIT*‐mutated GISTs from the stomach clustered in antrum, corpus, and fundus subgroups, suggesting regional epigenetic differences. Regional differences have been reported in gastric GISTs, such as epithelioid GISTs being more common in the antrum, while clinically malignant variants are more frequently found in the upper parts of the gastric body [4]. It is well established that GISTs arise from the interstitial cells of Cajal (ICC) or their stem cell precursors, and at least four distinct subpopulations of ICC have been identified, with specific distributions in the gastrointestinal tract [34]. The regional epigenetic differences may reflect diverse ICC‐lineage subpopulations from which GISTs arise, influencing tumor biology and progression.

In recent years, machine learning‐based classifiers developed at the German Cancer Research Centre (DKFZ) in Heidelberg have demonstrated the potential of DNA methylation‐based classification to improve diagnostics for central nervous system tumors and sarcomas [23, 35]. The DKFZ sarcoma classifier includes methylation classes for only about 30% of soft tissue and bone tumors listed in the current WHO classification. Despite its potential, its use in routine pathology diagnostics is still limited [36]. Supervised methylome classifiers can only recognize lesions of which methylation classes have been established in the underlying ground‐truth dataset. The DKFZ classifier correctly identified 24 out of 28 cases as GIST with calibrated scores > 0.9, indicating high classification accuracy. However, three cases with lower tumor cell content had calibrated scores between 0.3 and 0.9, and one SDH‐deficient GIST received no match (calibrated score < 0.3). The reduced recognition of the first three cases is likely attributable to their lower tumor cell percentage, a known factor that can affect individual clustering [37]. In contrast, the SDH‐deficient GIST had a high tumor cell content but still failed classification. A study by Miettinen et al. similarly reported an SDH‐deficient GIST that was not recognized by the classifier [25]. It is well documented that SDH‐deficient GISTs have distinct DNA methylation patterns compared to *KIT*/*PDGFRA*‐mutated GISTs [33]. The sarcoma classifier's inability to recognize SDH‐deficient GISTs may suggest that these tumors form a unique methylation class, for which the current classifier version lacks sufficient training data.

The progression from preclinical microscopic GIST to clinically manifest tumors with malignant potential involves the stepwise inactivation of tumor suppressor genes. Consistent with this, our results showed that progressive GISTs harbored the highest CNV burden, followed by high‐risk GISTs without disease progression, while low‐risk GISTs had the fewest CNVs.

Interestingly, patient #16 diagnosed with a low‐risk GIST exhibited multiple CNVs—unlike the other low‐risk GISTs—including loss of chr. 1p and homozygous deletion of *CDKN2A/B*. This patient did not receive adjuvant imatinib therapy due to the tumor's grading and developed metastatic disease 21 months after surgery. Upon re‐evaluation (including assessment of mitotic index), the tumor remained classified as low‐risk according to Miettinen's criteria. This case indicates that molecular risk classification based on CNVs could improve conventional risk assessment in GISTs, which currently relies on tumor localization, size, and mitotic index. Larger cohort studies are needed to determine whether metastatic GISTs classified as low‐risk by Miettinen's criteria consistently harbor multiple CNVs. Additionally, it remains unclear how many CNVs can occur in a low‐risk GIST and whether a distinct threshold exists for reclassifying a tumor as high‐risk.

Recently, Dermawan et al. proposed a three‐tier genomic risk stratification model for recurrence‐free survival [38]. In this model, gastric GISTs were graded as high‐risk if chr. 1p deletions were present and moderate‐risk if chr. 14q deletions were present or *KIT* exon 11 mutations were absent. Small bowel GISTs were graded as high‐risk if *MAX/MGA/MYC*, *CDKN2A*, or *RB1* alterations were present and moderate‐risk if chr. 1p deletions or chr. 5q amplifications were present. Applying this model, the gastric GIST of patient #16 would have been graded as high‐risk due to chr. 1p loss and homozygous deletion of *CDKN2A*.

In this exploratory study, we have analyzed DNA methylation and CNV patterns in a cohort of patients with GIST. Despite the relatively small sample size, our findings indicate that combining genome‐wide DNA methylation profiling with CNV analysis holds promise not only as a diagnostic tool but also as a molecular approach to improve GIST risk stratification. Future studies with larger cohorts, accounting for tumor location and genotype‐specific DNA methylation and CNV patterns, are necessary to validate these findings.

## Author Contributions

Tony G. Kleijn and Roos F. Bleckman conceptualized the study. Arjen H.G. Cleven and Anna K.L. Reyners supervised the study. Tony G. Kleijn and Baptiste Ameline analyzed the data. Tony G. Kleijn and Roos F. Bleckman drafted the manuscript and revised it. All the authors have read and approved the final manuscript.

## Ethics Statement

This study was approved by the medical ethic committee of the University Medical Centre Groningen (approval no. RR202200287). Tumor samples were coded (pseudonymized) according to the Code of Conduct for Health Research (www.coreon.org).

## Conflicts of Interest

The authors declare no conflicts of interest.

## Supporting information


**Figure S1.** Comparison of methylation of the *CLCN3* gene promotor between progressive and non‐progressive gastric gastrointestinal stromal tumors.

**Figure S2.** Average methylation levels of *CDKN2A*, *PAX3*, *REC8*, and *SPP1* in UMCG *KIT*/*PDGFRA*‐mutated gastrointestinal stromal tumors (*n* = 27).

**Figure S3.** Frequency of copy number variations (CNVs) in gastrointestinal stromal tumors (GISTs) located in the stomach (A) and small intestines (B). They were graded as low‐risk (C), moderate‐risk (D), and high‐risk (E) without progressive disease, or were associated with progressive disease (F), and had *KIT* (G) or *PDGFRA* mutations (H).

Table S1.

## Data Availability

The data generated in this study are available upon reasonable request from the corresponding author.
