# Supplementary figures and images for: Genome‐Wide DNA Methylation and Copy Number Alterations in Gastrointestinal Stromal Tumors
Source: Genes Chromosomes Cancer. 2025 Mar 27;64(3):e70046. doi: 10.1002/gcc.70046 (PMC11949093; doi:10.1002/gcc.70046)

Chromosome 4

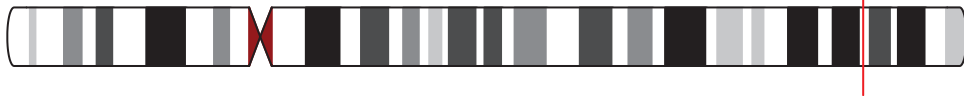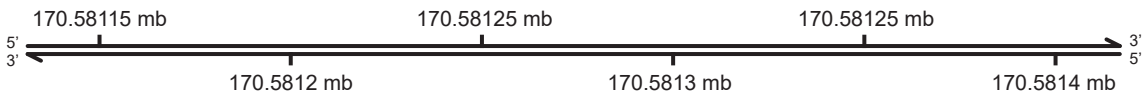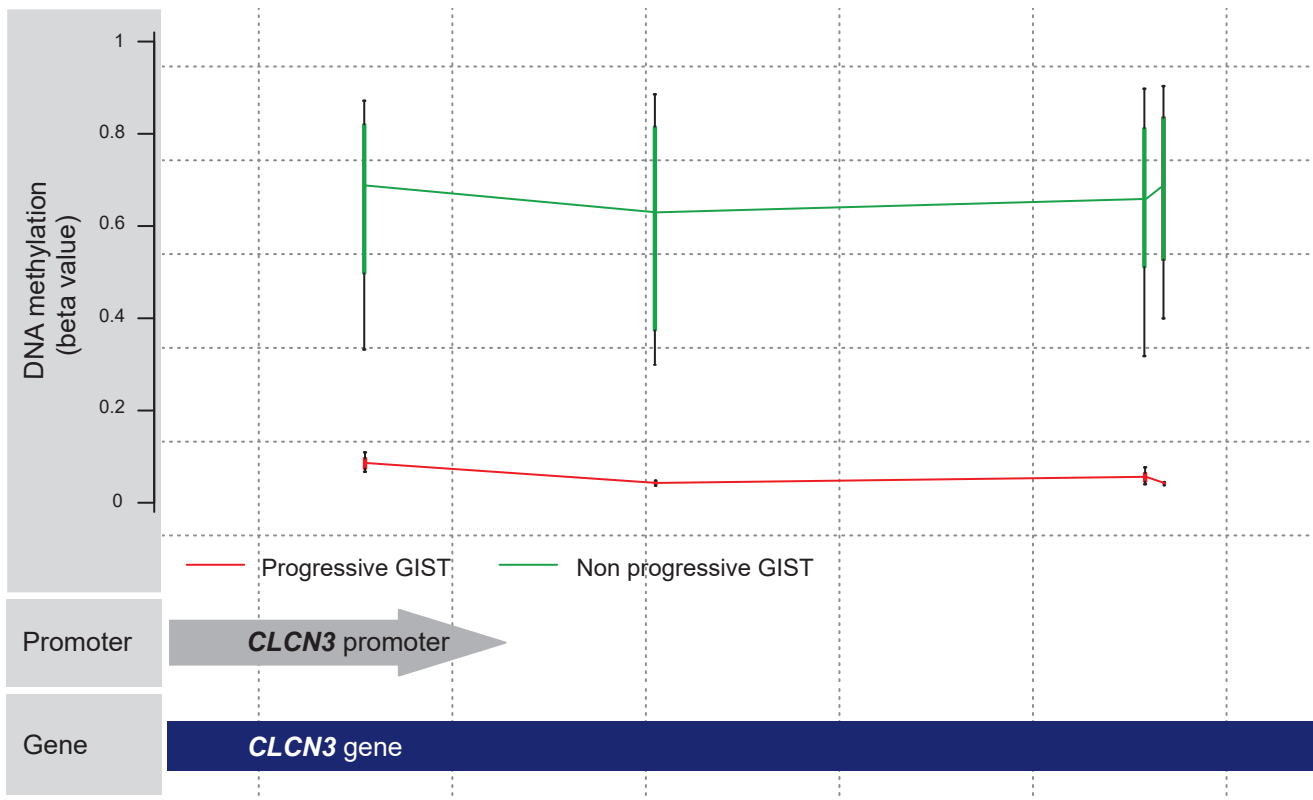

Supplement: Supplementary file 1 — Figure S1. Comparison of methylation of the CLCN3 gene promotor between progressive and non‐progressive gastric gastrointestinal stromal tumors. [file GCC-64-e70046-s004.pdf]

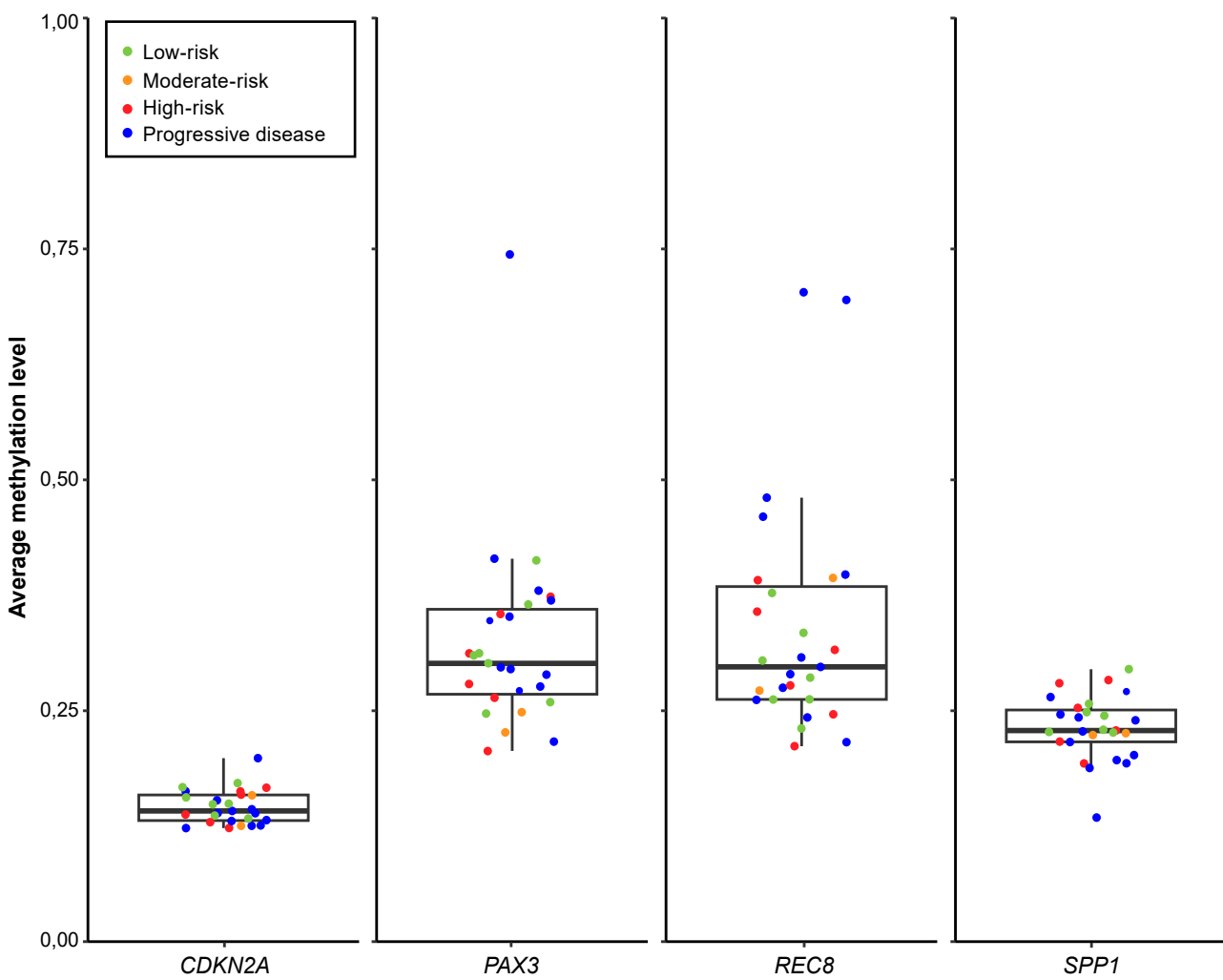

Supplement: Supplementary file 2 — Figure S2. Average methylation levels of CDKN2A, PAX3, REC8, and SPP1 in UMCG KIT/PDGFRA‐mutated gastrointestinal stromal tumors (n = 27). [file GCC-64-e70046-s001.pdf]

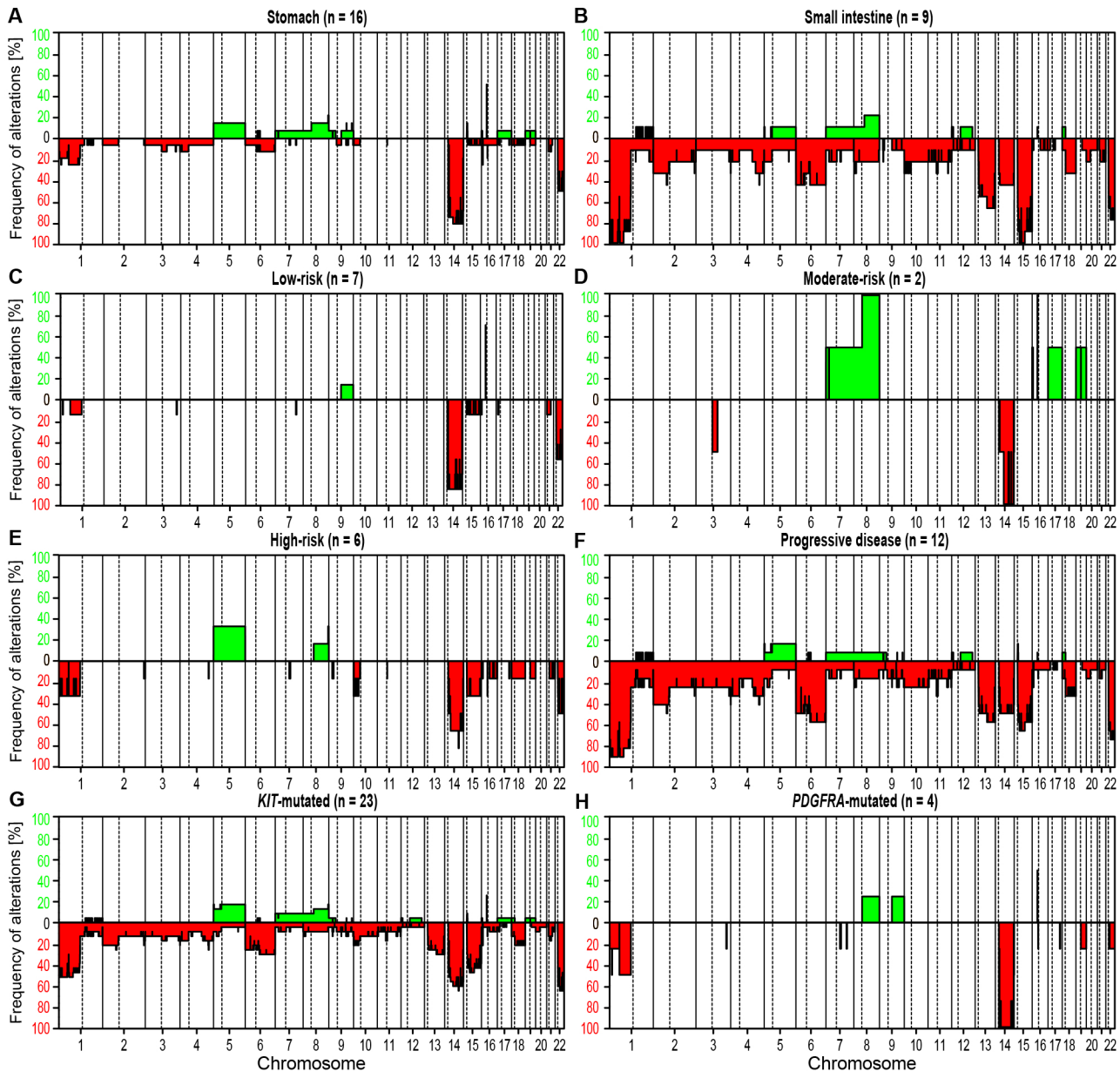

Supplement: Supplementary file 3 — Figure S3. Frequency of copy number variations (CNVs) in gastrointestinal stromal tumors (GISTs) located in the stomach (A) and small intestines (B). They were graded as low‐risk (C), moderate‐risk (D), and high‐risk (E) without progressive disease, or were associated with progressive disease (F), and had KIT (G) or PDGFRA mutations (H). [file GCC-64-e70046-s002.pdf]
