# Supplementary material for: Genome‐Wide DNA Methylation and Copy Number Alterations in Gastrointestinal Stromal Tumors
Source: Genes Chromosomes Cancer. 2025 Mar 27;64(3):e70046. doi: 10.1002/gcc.70046 (PMC11949093; doi:10.1002/gcc.70046)
Supplement: Supplementary file 4 — Table S1. [file GCC-64-e70046-s003.docx]

**Supplementary Table 1.**

Abbreviations: GIST: gastro-intestinal stromal tumor; KRYO: fresh-frozen tumor tissue; FFPE: formalin-fixed paraffin-embedded tumor tissue; F: female, M: male, NA: not available.

| **No.** | **Sex** | **Tumor site** | **Driver gene** | **Heidelberg classifier** | **Sample type** | **Array type** | **Sentrix ID** | **Reference** |
| --- | --- | --- | --- | --- | --- | --- | --- | --- |
| 1 | F | Stomach (fundus) | *KIT* | GIST, calibrated score: 99 | KRYO | EPIC | 206848800138_R01C01 | - |
| 2 | F | Stomach (fundus) | *PDGFRA* | GIST, calibrated score: 99 | KRYO | EPIC | 206848800138_R02C01 | - |
| 3 | F | Stomach (corpus) | *KIT* | GIST, calibrated score: 99 | KRYO | EPIC | 206848800138_R03C01 | - |
| 4 | F | Stomach (corpus) | *KIT* | GIST, calibrated score: 99 | KRYO | EPIC | 206848800138_R04C01 | - |
| 5 | M | Stomach (fundus) | *KIT* | GIST, calibrated score: 99 | KRYO | EPIC | 206848800138_R05C01 | - |
| 6 | F | Stomach (fundus) | *KIT* | GIST, calibrated score: 99 | KRYO | EPIC | 206848800138_R06C01 | - |
| 7 | M | Stomach (antrum) | *KIT* | GIST, calibrated score: 99 | KRYO | EPIC | 206848800138_R07C01 | - |
| 8 | M | Stomach (antrum) | *KIT* | GIST, calibrated score: 99 | KRYO | EPIC | 206848800078_R06C01 | - |
| 9 | F | Stomach (corpus) | *PDGFRA* | GIST, calibrated score: 99 | KRYO | EPIC | 206848800078_R02C01 | - |
| 10 | F | Stomach (corpus) | *PDGFRA* | GIST, calibrated score: 99 | KRYO | EPIC | 206848800078_R03C01 | - |
| 11 | F | Stomach (antrum) | *PDGFRA* | GIST, calibrated score: 99 | KRYO | EPIC | 206848800078_R04C01 | - |
| 12 | M | Stomach (corpus) | *KIT* | GIST, calibrated score: 99 | KRYO | EPIC | 206848800078_R05C01 | - |
| 13 | F | Stomach (fundus) | *KIT* | GIST, calibrated score: 44 | KRYO | EPIC | 206848800138_R08C01 | - |
| 14 | F | Small intestine | *KIT* | GIST, calibrated score: 34 | KRYO | EPIC | 206848800078_R07C01 | - |
| 15 | F | Ileum | *KIT* | GIST, calibrated score: 99 | KRYO | EPIC | 206848800078_R08C01 | - |
| 16 | M | Stomach (corpus) | *KIT* | GIST, calibrated score: 95 | KRYO | EPIC | 206848800084_R02C01 | - |
| 17 | F | Small intestine | *KIT* | GIST, calibrated score: 99 | KRYO | EPIC | 206848800084_R05C01 | - |
| 18 | F | Small intestine | *KIT* | GIST, calibrated score: 99 | KRYO | EPIC | 206848800084_R06C01 | - |
| 19 | F | Stomach (corpus) | *KIT* | GIST, calibrated score: 99 | KRYO | EPIC | 206848800084_R04C01 | - |
| 20 | M | Stomach (corpus) | *KIT* | GIST, calibrated score: 99 | KRYO | EPIC | 206848800084_R01C01 | - |
| 21 | M | Ileum | *KIT* | GIST, calibrated score: 99 | FFPE | EPIC | 205975520070_R02C01 | - |
| 22 | M | Small intestine | *KIT* | GIST, calibrated score: 99 | KRYO | EPIC | 206848800084_R07C01 | - |
| 23 | M | Jejunum | *KIT* | GIST, calibrated score: 99 | KRYO | EPIC | 206848800084_R08C01 | - |
| 24 | M | Ileum | *KIT* | GIST, calibrated score: 99 | KRYO | EPIC | 206848800085_R01C01 | - |
| 25 | M | Ileum | *KIT* | GIST, calibrated score: 99 | KRYO | EPIC | 206848800085_R02C01 | - |
| 26 | M | Ileum | *KIT* | GIST, calibrated score: 94 | KRYO | EPIC | 206848800085_R03C01 | - |
| 27 | M | Rectum | *KIT* | GIST, calibrated score: 63 | KRYO | EPIC | 206848800085_R04C01 | - |
| 28 | M | Stomach | *SDHA* | GIST, calibrated score: 0 | KRYO | EPIC | 206848800084_R03C01 | - |
| 29 | M | Stomach | NA | Reference case of GIST cluster | FFPE | EPIC | 200514030106_R08C01 | [23] |
| 30 | M | Stomach | NA | Reference case of GIST cluster | FFPE | EPIC | 200514030106_R07C01 | [23] |
| 31 | M | Stomach | NA | Reference case of GIST cluster | FFPE | EPIC | 200514030106_R05C01 | [23] |
| 32 | M | Stomach | NA | Reference case of GIST cluster | FFPE | EPIC | 200514030106_R04C01 | [23] |
| 33 | M | Stomach | NA | Reference case of GIST cluster | FFPE | EPIC | 200514030106_R03C01 | [23] |
| 34 | M | Stomach | NA | Reference case of GIST cluster | FFPE | EPIC | 200514030106_R02C01 | [23] |
| 35 | F | Omentum | NA | Reference case of GIST cluster | FFPE | EPIC | 200514030106_R01C01 | [23] |
| 36 | M | Stomach | NA | Reference case of GIST cluster | FFPE | EPIC | 200550900084_R08C01 | [23] |
| 37 | M | Small intestine | NA | Reference case of GIST cluster | FFPE | EPIC | 200550900084_R07C01 | [23] |
| 38 | F | Stomach | NA | Reference case of GIST cluster | FFPE | EPIC | 200550900084_R06C01 | [23] |
| 39 | M | Small intestine | NA | Reference case of GIST cluster | FFPE | EPIC | 200550900084_R05C01 | [23] |
| 40 | M | Small intestine | NA | Reference case of GIST cluster | FFPE | EPIC | 200550900084_R04C01 | [23] |
| 41 | F | Small intestine | NA | Reference case of GIST cluster | FFPE | EPIC | 200550900084_R03C01 | [23] |
| 42 | M | Stomach | NA | Reference case of GIST cluster | FFPE | EPIC | 200550900084_R02C01 | [23] |
| 43 | M | Stomach | NA | Reference case of GIST cluster | FFPE | EPIC | 200550900061_R08C01 | [23] |
| 44 | F | Stomach | NA | Reference case of GIST cluster | FFPE | EPIC | 200550900061_R07C01 | [23] |
| 45 | F | Small intestine | NA | Reference case of GIST cluster | FFPE | EPIC | 200550900061_R06C01 | [23] |
| 46 | M | Stomach | NA | Reference case of GIST cluster | FFPE | EPIC | 200550900061_R05C01 | [23] |
| 47 | F | Stomach | NA | Reference case of GIST cluster | FFPE | EPIC | 200550900061_R04C01 | [23] |
| 48 | M | Small intestine | NA | Reference case of GIST cluster | FFPE | EPIC | 200550900061_R03C01 | [23] |
| 49 | F | Small intestine | NA | Reference case of GIST cluster | FFPE | EPIC | 200550900061_R01C01 | [23] |
| 50 | M | Stomach | NA | Reference case of GIST cluster | FFPE | EPIC | 200550900090_R08C01 | [23] |
| 51 | M | Stomach | NA | Reference case of GIST cluster | FFPE | EPIC | 200550900090_R05C01 | [23] |
| 52 | M | Stomach | NA | Reference case of GIST cluster | FFPE | EPIC | 200550900090_R03C01 | [23] |
| 53 | F | Stomach | NA | Reference case of GIST cluster | FFPE | EPIC | 200550900090_R02C01 | [23] |
| 54 | M | Stomach | NA | Reference case of GIST cluster | FFPE | EPIC | 200550900090_R01C01 | [23] |
| 55 | F | Stomach | NA | Reference case of GIST cluster | FFPE | EPIC | 200550900073_R07C01 | [23] |
| 56 | M | Stomach | NA | Reference case of GIST cluster | FFPE | EPIC | 200550900073_R04C01 | [23] |
| 57 | M | Stomach | NA | Reference case of GIST cluster | FFPE | EPIC | 200550900073_R06C01 | [23] |
| 58 | F | Stomach | NA | Reference case of GIST cluster | FFPE | EPIC | 200550900084_R01C01 | [23] |
| 59 | M | Sigma | NA | Reference case of GIST cluster | FFPE | EPIC | 200550900073_R01C01 | [23] |
| 60 | F | Rectum | NA | Reference case of GIST cluster | FFPE | EPIC | 200514030106_R06C01 | [23] |
| 61 | F | Stomach | NA | Reference case of GIST cluster | FFPE | EPIC | 200550900073_R05C01 | [23] |
| 62 | M | Small intestine | NA | Reference case of GIST cluster | FFPE | EPIC | 200550900073_R02C01 | [23] |
| 63 | NA | Lower limbs | *PDGFRA* | GIST, calibrated score: 99 | KRYO | EPIC | 201465950033_R07C01 | [23] |
| 64 | NA | Pelvis | NA | GIST, calibrated score: 99 | KRYO | EPIC | 201465950030_R08C01 | [23] |
| 65 | NA | Small intestine | NA | GIST, calibrated score: 92 | KRYO | EPIC | 201465950030_R06C01 | [23] |
| 66 | NA | Small intestine | NA | GIST, calibrated score: 99 | KRYO | EPIC | 201465950027_R04C01 | [23] |
| 67 | F | Stomach | SDH-deficient | NA | FFPE | EPIC | 205107400076_R03C01 | [25] |
| 68 | M | Liver | SDH-deficient | NA | FFPE | EPIC | 206537460031_R06C01 | [25] |
